# Effectiveness of Text Messaging Interventions on BMI Among Adults With Prediabetes: Systematic Review and Meta-Analysis

**DOI:** 10.2196/78521

**Published:** 2026-04-30

**Authors:** Arkers Kwan Ching Wong, Shun Yan Choi, Tiffany Tsz Yan Cheng, Chelsea Cheuk Yiu Cheng, Ivy Yuk Man Kwong, Cheuk Yin Fong, Chi Sum Wong, Ka Lo Chan

**Affiliations:** 1 School of Nursing, Hong Kong Polytechnic University, GH 502, 1 Cheong Wan Road, Hung Hom, China (Hong Kong), 852 34003805; 2 Joint Research Centre for Primary Health Care, Hong Kong Polytechnic University, Hong Kong, China (Hong Kong)

**Keywords:** prediabetes, text messages, meta-analysis, telehealth, mobile health, Preferred Reporting Items for Systematic Reviews and Meta-Analyses, PRISMA

## Abstract

**Background:**

Prediabetes is a growing global health concern. Lifestyle modification is the cornerstone of management, yet scalable delivery strategies are needed. SMS text messaging is a promising mobile health approach for behavior change, but its effectiveness for metabolic outcomes in prediabetes remains uncertain.

**Objective:**

This study aims to evaluate the effectiveness of text message–delivered lifestyle programs for BMI among adults with prediabetes and, secondarily, for weight, waist circumference, hemoglobin A_1c_ (HbA_1c_), total cholesterol, and diabetes incidence.

**Methods:**

We conducted a PRISMA (Preferred Reporting Items for Systematic Reviews and Meta-Analyses)-guided systematic review and meta-analysis of randomized controlled trials (searching the PubMed, MEDLINE, CINAHL, Embase, Web of Science, and the Cochrane Library databases; from April 2005 to March 2025). Three reviewers independently screened studies, extracted data, and assessed risk of bias (using the Cochrane Risk of Bias tool), and random-effects meta-analyses were performed.

**Results:**

In total, 7 randomized controlled trials (n=4632) met the inclusion criteria. SMS text messaging did not significantly affect BMI compared with standard care (mean difference [MD] −0.17 kg/m², 95% CI −0.85 to 0.25; *I*²=32%). Secondary outcomes were similarly nonsignificant: weight (MD −0.46 kg, 95% CI −1.74 to 0.83; *I*²=27%); waist circumference (MD −0.36 cm, 95% CI −1.09 to 0.36; *I*²=13%); HbA_1c_ (MD −0.05%, 95% CI −0.17 to 0.07; *I*²=89%); total cholesterol (standardized MD −0.00, 95% CI −0.06 to 0.06; *I*²=0%); and diabetes incidence (odds ratio 0.84, 95% CI 0.63-1.12, *I*²=51%; n=3515 across 3 trials). Estimates were small and, for several outcomes, notably HbA_1c_ and diabetes incidence, were imprecise and heterogeneous, indicating substantial between-study variability.

**Conclusions:**

In adults with prediabetes, lifestyle programs incorporating SMS text messaging showed no meaningful effect on BMI or other metabolic outcomes; pooled estimates were near null with considerable uncertainty and heterogeneity for some end points. Variability in intervention design, dose, and tailoring likely contributed to these results. Future trials should extend follow-up, report body composition outcomes in addition to BMI, and test tailored, interactive, closed-loop SMS text messaging strategies with adequate power to resolve heterogeneous effects.

## Introduction

Prediabetes affects approximately 374 million adults aged 20 to 79 years worldwide and is projected to increase to 730 million by 2045 [1,2]. It is defined by impaired fasting glucose or impaired glucose tolerance, with blood glucose levels elevated but below the threshold for a type 2 diabetes diagnosis [3,4]. Prediabetes is associated with increased risks of cardiovascular disease, microvascular complications, and all-cause mortality, and approximately 5% to 10% of individuals with prediabetes progress to type 2 diabetes annually [1]. Due to its asymptomatic nature, prediabetes often remains undiagnosed until significant disease onset, underscoring the need for early preventive strategies.

Lifestyle modification is the first-line approach for prediabetes management, demonstrating efficacy in delaying or preventing type 2 diabetes [5]. Compared with pharmacological interventions, lifestyle programs are preferred by individuals and focus on dietary changes, increased physical activity, and behavioral support [6]. Adopting healthy eating patterns, such as the Mediterranean diet, improves glycemic control [7], while enhanced physical activity improves insulin sensitivity and reduces diabetes risk [8]. Behavioral strategies, including goal setting and self-monitoring, are critical for sustaining lifestyle changes [9]. Given that an elevated BMI is a strong risk factor for diabetes progression, targeting BMI through lifestyle interventions is central to prediabetes prevention efforts [10].

Mobile health (mHealth) technologies have emerged as scalable tools for delivering lifestyle interventions. Numerous studies have demonstrated that mHealth interventions, including mobile apps, wearable devices, and SMS text messaging, can effectively support behavior change and reduce the incidence of type 2 diabetes [11-14]. Among these mHealth interventions, SMS text messaging stands out for its simplicity. SMS text messaging, in particular, is widely accessible, cost-effective, and convenient, leveraging ubiquitous mobile phone use to promote health behaviors [15-17]. Messages can be retained, accessed flexibly, and tailored to individual needs, which may enhance engagement and adherence [11-14].

SMS text messaging interventions, guided by behavior change theories such as self-determination theory, offer motivational support and personalized feedback that facilitate sustainable behavior change [18-20]. Compared with other mHealth modalities, SMS text messaging imposes minimal user burden and provides an efficient, scalable approach to lifestyle modification [21]. These advantages are particularly relevant for individuals with prediabetes, who often face barriers to program participation, including socioeconomic constraints, time limitations, and low motivation [16,22].

Despite growing interest in SMS text messaging interventions for lifestyle modification, evidence specific to adults with prediabetes remains limited. Prior reviews have predominantly focused on populations with diagnosed diabetes or obesity, which may differ in baseline awareness, motivation, and response to interventions. As prediabetes continues to rise globally, understanding the effectiveness of SMS text messaging–based interventions in this specific population is critical.

This systematic review and meta-analysis aimed to evaluate the effectiveness of lifestyle modification programs using SMS text messaging on BMI reduction among adults with prediabetes, compared with standard care. Secondary outcomes included changes in weight, waist circumference, hemoglobin A_1c_ (HbA_1c_), total cholesterol, and incidence of diabetes. By synthesizing available evidence, this study addresses the lack of targeted evidence for digital interventions in prediabetes management, a distinct group often underrepresented in diabetes prevention research. The findings are expected to directly inform the development of mHealth strategies tailored to the population with prediabetes, optimizing early intervention efforts. Furthermore, by identifying the role of SMS text messaging in BMI reduction and metabolic improvements, this review provides a foundation for implementing scalable, low-cost digital solutions to curb diabetes progression at both clinical and public health levels.

## Methods

### Overview

This systematic review and meta-analysis was conducted in accordance with the PRISMA (Preferred Reporting Items for Systematic Reviews and Meta-Analyses) 2020 guidelines (Checklist 1). It was registered in PROSPERO with the number CRD420251055972. A protocol for this systematic review was not prepared.

### Search Strategy

A comprehensive literature search was performed by 3 independent reviewers (TTYC, CYF, and AKCW) across 6 electronic databases: PubMed, MEDLINE, CINAHL, Embase, Web of Science, and the Cochrane Library. Studies published between April 1, 2005, and March 31, 2025, were included. The reference lists of included studies were also hand-searched independently by the 3 reviewers (TTYC, CYF, and AKCW) to identify additional eligible studies. Any disagreements among the reviewers were resolved through discussion.

Only peer-reviewed articles were considered; gray literature and review articles were excluded to ensure the inclusion of high-quality evidence. Search strategies were developed using key terms including “prediabetes” and “text messaging” and were expanded using Medical Subject Headings (MeSH) terms where appropriate. Detailed search strategies for each database are provided in Multimedia Appendix 1.

### Study Selection

Studies were eligible for inclusion if they met the following criteria: randomized controlled trials (RCTs), participants aged ≥18 years diagnosed with prediabetes, interventions consisting of lifestyle modification programs (ie, diet control, exercise, or behavioral therapy), and use of text messages—defined as SMS, multimedia messaging service, or instant messaging via mobile apps—as the primary intervention delivery channel.

The exclusion criteria were as follows: participants diagnosed with type 2 diabetes mellitus (DM) or gestational diabetes, participants taking medications known to affect glucose tolerance, and studies without complete analytical outcome data.

### Outcome

The primary outcome of interest was change in BMI. Secondary outcomes included changes in body weight, waist circumference, HbA_1c_, total cholesterol, and incidence of DM.

### Quality Assessment and Risk of Bias

The risk of bias for the included studies was assessed using the Risk of Bias tool (Cochrane Collaboration). This tool evaluates the following five domains: (1) bias arising from the randomization process, (2) bias due to deviations from intended interventions, (3) bias due to missing outcome data, (4) bias in the measurement of outcomes, and (5) bias in the selection of reported results. Any disagreements among the 3 reviewers were resolved through discussion. As there were fewer than 10 included studies, a funnel plot to assess publication bias was not constructed.

### Certainty of Evidence (Grading of Recommendations, Assessment, Development, and Evaluation)

Beyond study-level risk of bias, we appraised the outcome-level certainty of evidence using the GRADE (Grading of Recommendations Assessment, Development and Evaluation) approach for each meta-analyzed outcome (BMI, body weight, waist circumference, HbA_1c_, total cholesterol, and diabetes incidence). Because all included studies were RCTs, each outcome began at high certainty and could be rated down for risk of bias, inconsistency, indirectness, imprecision, and publication bias. The domains were assessed as follows:

- Risk of bias; considered the risk of bias domain ratings contributing to each outcome
- Inconsistency; considered heterogeneity (*I*² and between-study variability) and overlap of study CIs
- Indirectness; assessed population, intervention, comparator, and outcome alignment with the review question
- Imprecision; considered 95% CIs (including whether they crossed the line of no effect) and optimal information size
- Publication bias; formal tests were not performed given <10 studies; we qualitatively considered small-study effects and selective reporting.

We present GRADE ratings and explicit downgrading rationales in a summary of findings table (Table S1 in Multimedia Appendix 2) and detailed footnotes (Multimedia Appendix 2). No outcomes were upgraded.

### Data Extraction

Data from the included studies were retrieved and independently entered into a Microsoft Excel spreadsheet by 3 independent reviewers (TTYC, CYF, and AKCW). Additional variables—including author, study location, study population, study duration, intervention and control details, data collection time points, outcome measures, and results—were also extracted from the selected studies (Multimedia Appendix 3).

### Data Synthesis and Analysis

Meta-analyses were conducted using Review Manager (version 5.4.1; Cochrane Collaboration). Outcomes were analyzed at the longest reported follow-up time point and illustrated by forest plots. For continuous variables, mean differences (MDs) with 95% CIs were calculated under a random-effects model. The meta-analysis was conducted using the postintervention values reported by the individual studies. For dichotomous variables, odds ratios and 95% CIs were computed using the inverse variance method.

Statistical heterogeneity was assessed using the chi-square test, the *I*² statistic, and associated *P* values (*P*<.05 was considered statistically significant). *I*² values were interpreted as follows: unimportant (0%‐40%), moderate (30%‐60%), substantial (50%‐90%), or considerable (75%‐100%) heterogeneity. Given the limited number of included trials (<10), subgroup analyses, meta-regression, and sensitivity analyses were not conducted.

## Results

### Study Selection

**Figure 1.**
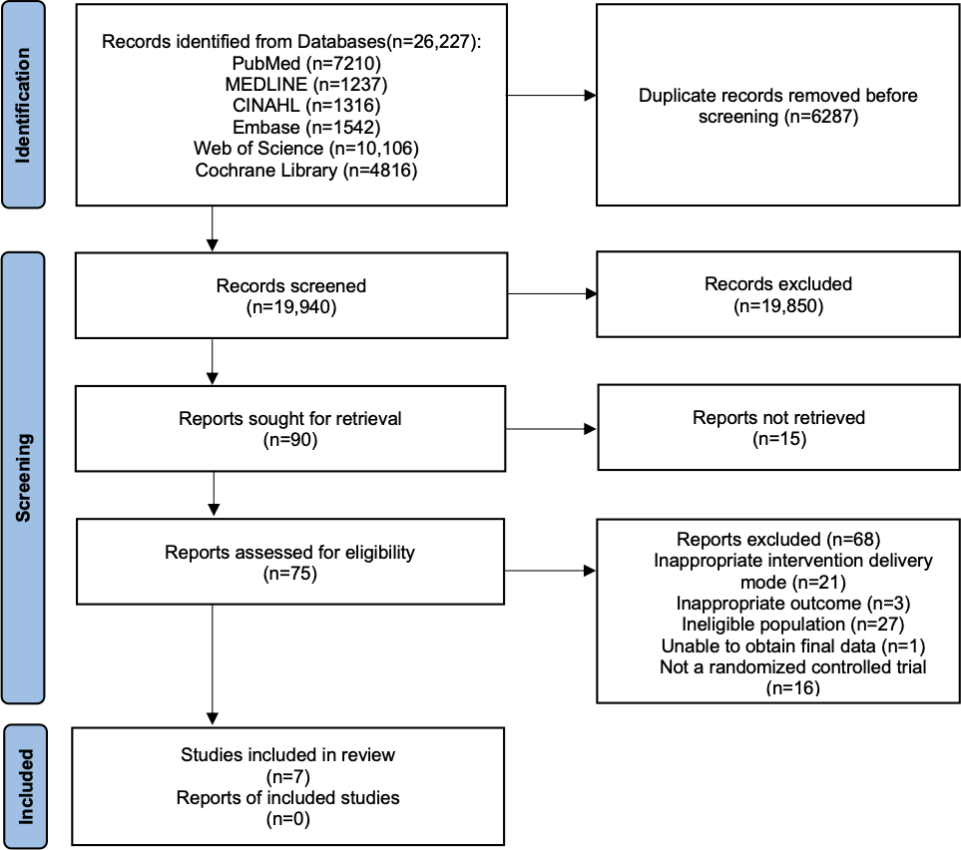
PRISMA (Preferred Reporting Items for Systematic Reviews and Meta-Analyses) 2020 flowchart.

A total of 19,940 publications were identified after duplicate removal. Title and abstract screening excluded 19,850 (99.5%) publications. Of the remaining 90 publications, 75 (83.3%) were available for full-text review and assessed for eligibility. In total, 68 (90.7%) publications were excluded, primarily due to inappropriate intervention delivery modes, ineligible populations, or study designs other than RCTs. Only 7 (9.4%) publications met the eligibility criteria and were included in this review [16,23-28] (Figure 1).

### Risk of Bias Assessment

Risk of bias among the 7 included RCTs, assessed using the Cochrane Risk of Bias tool, ranged from “some concerns” to “high risk.” All included studies demonstrated low risk in the domain “bias in selection of the reported result.” However, 3 of 7 included studies were rated as “some concerns” for the domains of bias arising from the randomization process, missing outcome data, and measurement of outcomes. All included studies were rated as “some concerns” for bias due to deviations from intended interventions, likely reflecting the nonblinded nature of the interventions. A summary of the risk of bias assessments is presented in Table 1.

**Table 1.** Risk of bias of the included studies.

Study	Randomization process	Deviation from intended intervention	Missing outcome data	Measurement of outcomes	Selection of the reported result	Overall risk of bias
Al-Hamdan et al [26]	Some concerns	Some concerns	Low	Low	Low	Some concerns
Bootwong and Intarut [16]	Some concerns	Some concerns	Low	Low	Low	Some concerns
Chung et al [27]	Low	Some concerns	Some concerns	Some concerns	Low	High
Khunti et al [28]	Low	Some concerns	Some concerns	Some concerns	Low	High
Nanditha et al [23]	Low	Some concerns	Some concerns	Low	Low	Some concerns
Ramachandran et al [24]	Low	Some concerns	Low	Some concerns	Low	Some concerns
Wong et al [25]	Some concerns	Some concerns	Some concerns	Low	Low	High

### Certainty of Evidence (GRADE) Summary

Outcome-level certainty ratings and rationales are reported in Table S1 (summary of findings) in Multimedia Appendix 2. In brief, certainty was most commonly rated down for inconsistency (between-study heterogeneity) and imprecision (wide CIs and limited sample size per outcome). Publication bias could not be formally assessed due to fewer than 10 studies, and no outcomes were upgraded.

### Characteristics of Participants and Studies

The 7 included RCTs enrolled a total of 4632 participants diagnosed with prediabetes. All participants owned smartphones and were free of major comorbidities. The mean age of participants ranged from 43.5 to 54.2 (SD 5.6) years. Of 4632 participants, 2843 (61.4%) were male.

Among the participants, 2056 (44.4%) individuals received lifestyle modification programs incorporating SMS text messaging, while 2576 (55.6%) individuals received comparable programs without SMS text messaging support. Intervention durations ranged from 12 to 48 months, with 24 months (n=2) and 48 months (n=2) being the most common durations. Delivery formats varied: 4 studies delivered regular text messages promoting lifestyle modification [16,23-25], 2 studies combined mHealth apps with SMS text messaging support [26,27], and 1 study integrated face-to-face sessions with text message follow-ups [28]. For the control group, usual care included related education (n=4), paper-based health information materials (n=2), and physical activity management (n=1). Characteristics of all included studies are summarized in Multimedia Appendix 3.

### Quantitative Synthesis

**Figure 2.**
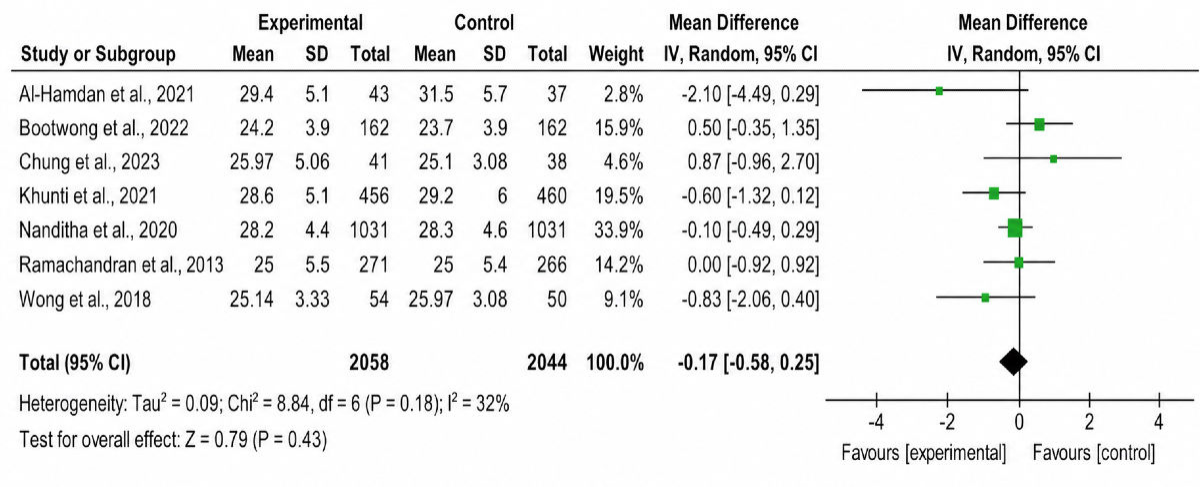
Forest plot of the effect of SMS text messaging interventions on BMI compared with standard care in adults with prediabetes: 1 primary outcome; outcome 1.1 BMI [16,23-28].

The forest plots of both primary and secondary outcomes are shown in Figures2^7^.

**Figure 3.**
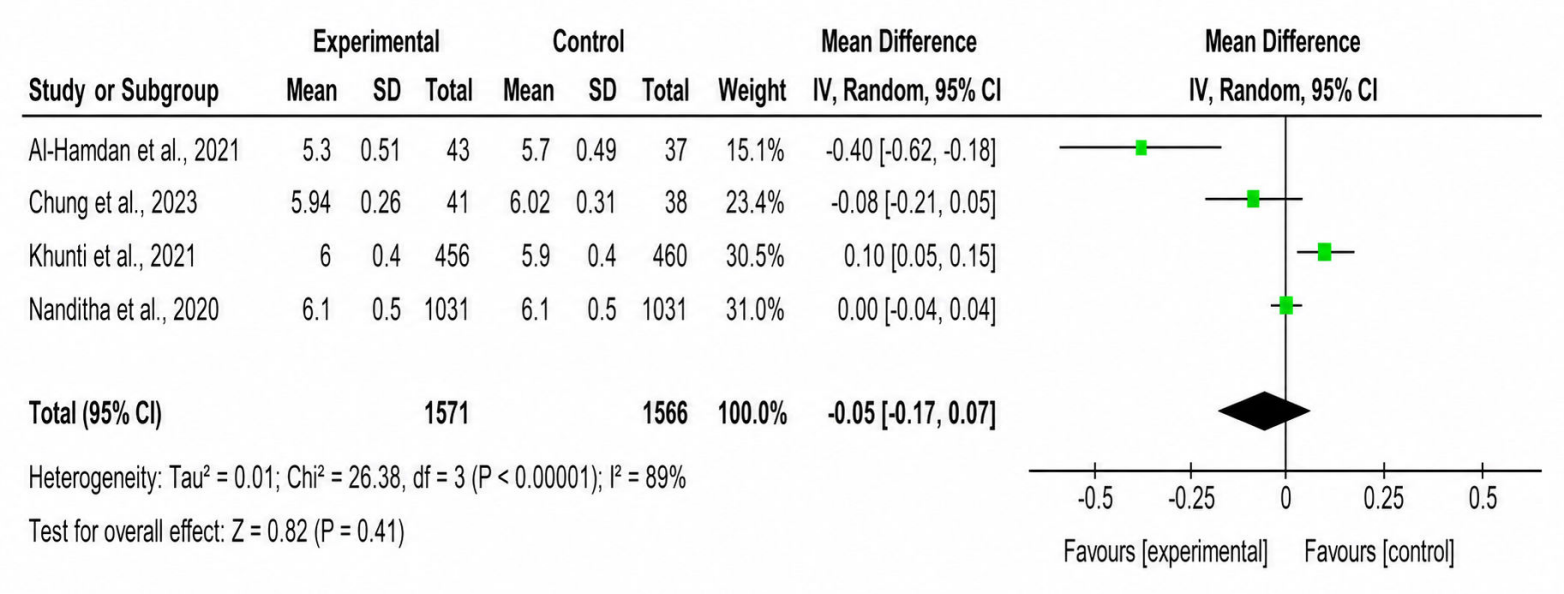
Forest plot of the effect of SMS text messaging interventions on hemoglobin A_1c_ compared with standard care in adults with prediabetes [23,26-28].

**Figure 4.**
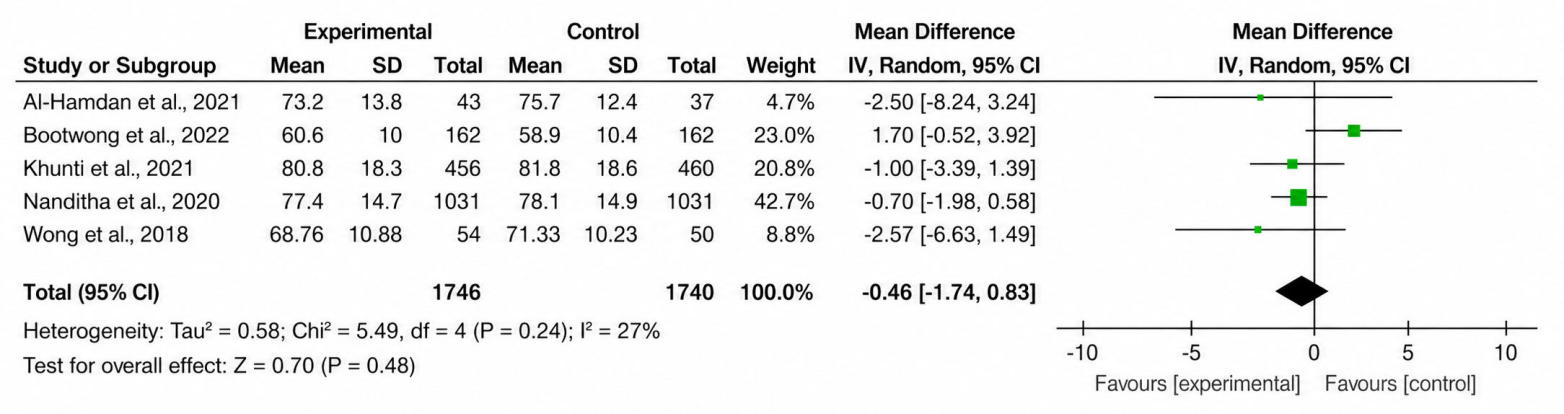
Forest plot of the effect of SMS text messaging interventions on body weight compared with standard care in adults with prediabetes [16,23-26,28].

**Figure 5.**
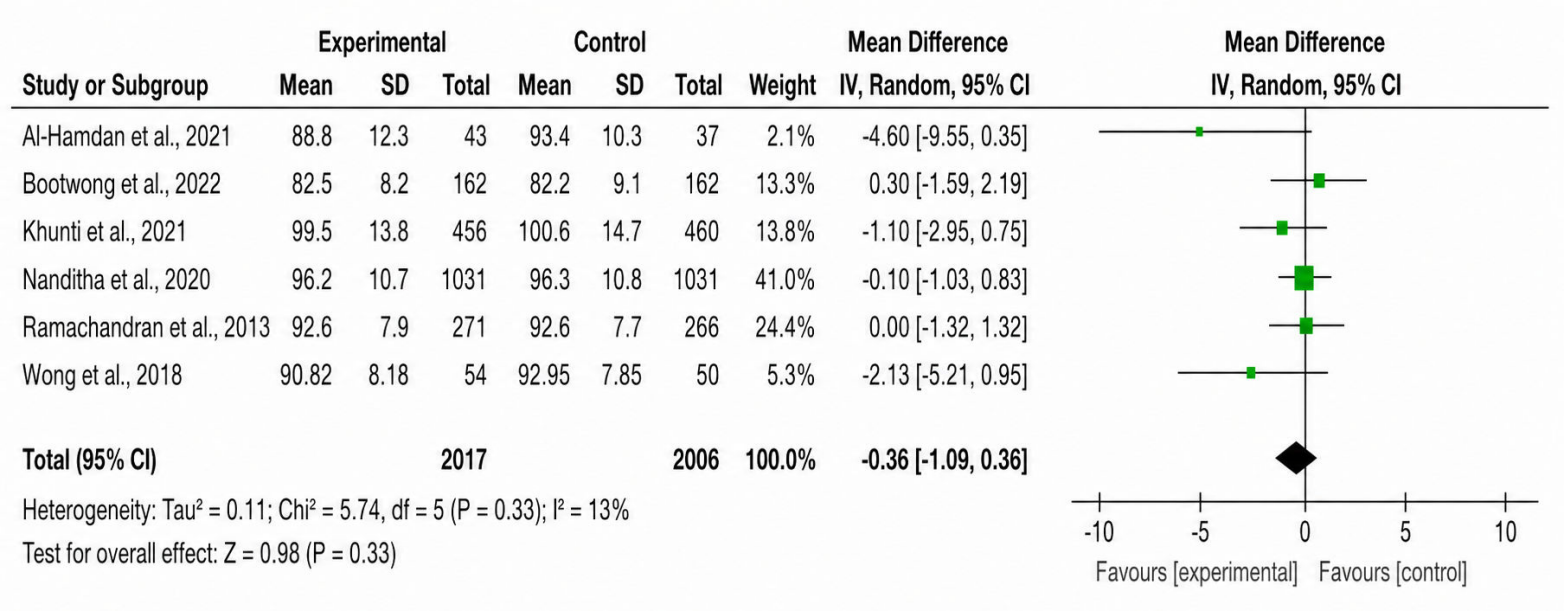
Forest plot of the effect of SMS text messaging interventions on waist circumference [16,23-26,28].

**Figure 6.**
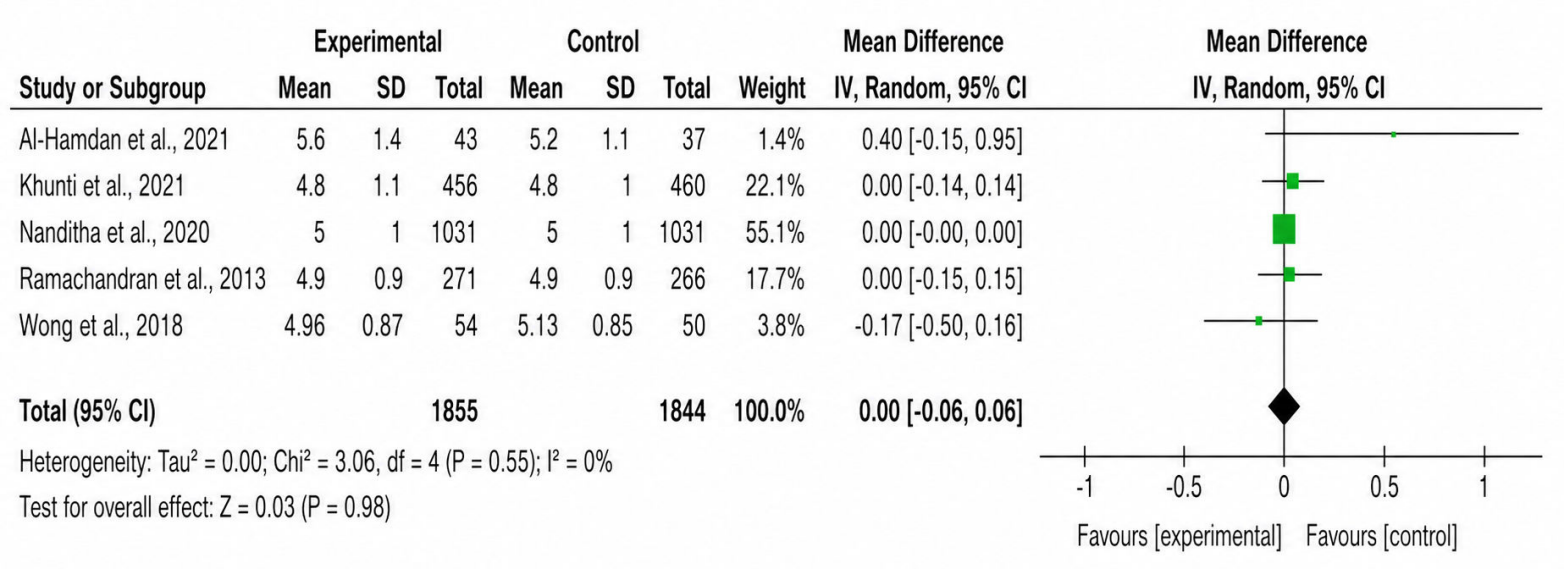
Forest plot of the effect of SMS text messaging interventions on total cholesterol [23-26,28].

**Figure 7.**
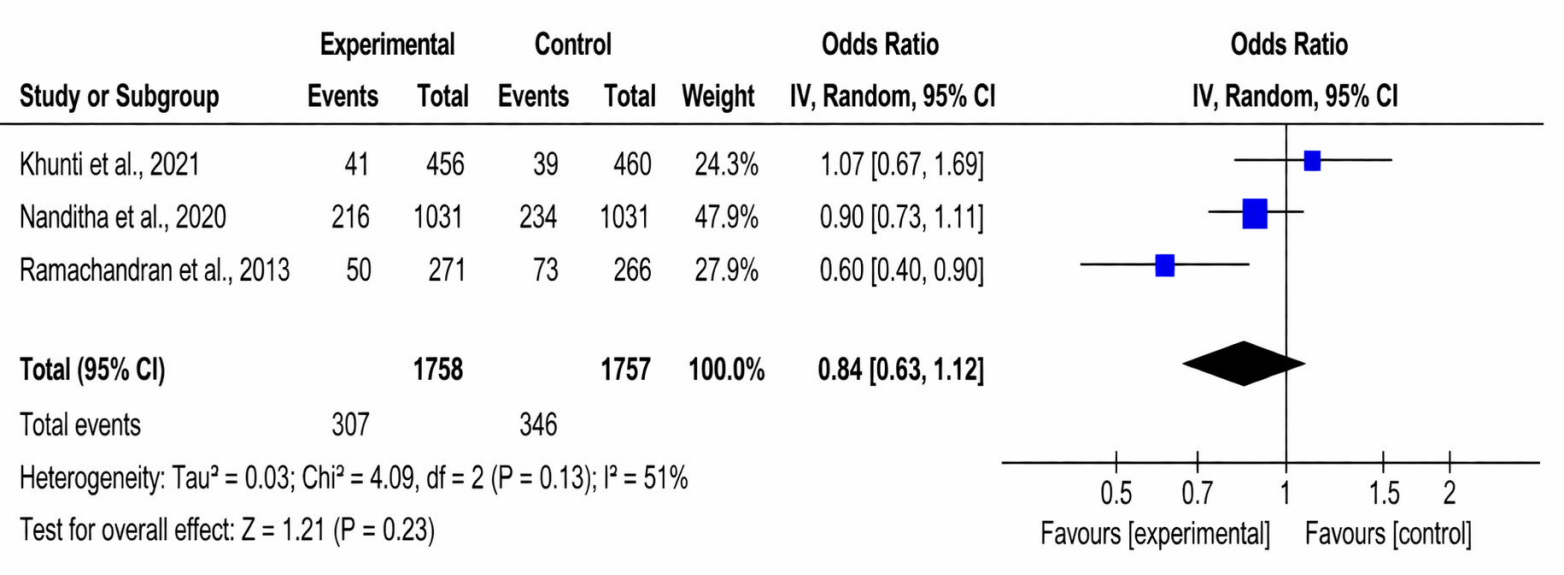
Forest plot of the effect of text messaging interventions on diabetes incidence [23,24,28].

### Primary Outcome: BMI

All 7 studies assessed BMI (4102/4634, 88.5% participants). The pooled MD in BMI change was not statistically significant between intervention and control groups (MD –0.17, 95% CI –0.85 to 0.25; *P*=.43), with low heterogeneity observed (*χ*²_6_=8.8; *I*²=32%; *P*=.18).

### Secondary Outcomes

#### Weight

Of the 7 studies, 5 (71.4%) reported weight outcomes. The pooled MD was –0.46 (95% CI –1.74 to 0.83; *P*=.48), indicating no significant difference in weight change between groups. Low heterogeneity was observed (*χ*²_5_=5.49; *I*²=27%; *P*=.24).

#### Waist Circumference

In total, 6 (85.7%) studies assessed waist circumference. The pooled MD was –0.36 (95% CI –1.09 to 0.36; *P*=.33), with low heterogeneity (*χ²*_5_=5.74, *I*²=13%; *P*<.001).

#### HbA_1c_

Overall, 4 studies assessed HbA_1c_ levels. Only 1 study reported a significant improvement favoring the SMS text messaging group. The pooled MD was –0.05 (95% CI –0.17 to 0.07; *P*=.41), with high heterogeneity across studies (*χ*²_3_=3.06, *I*²=89%; *P*=.49).

#### Total Cholesterol

A meta-analysis of 5 studies showed no significant difference in total cholesterol levels between the intervention and control groups (standardized MD –0.00, 95% CI –0.06 to 0.06; *P*=.98). No heterogeneity was observed (*χ*²_4_=18.2, *I*²=0%; *P*=.55).

#### DM Incidence

DM incidence was reported in 3 studies (3515/653, 18.6% participants). Moderate heterogeneity was noted (*χ*²_2_=4.09, *I*²=51%; *P*=.13). Among intervention participants, 307 (17.5%) of 1758 developed diabetes, compared with 346 (19.7%) of 1757 in the control group. The odds ratio for DM incidence was 0.84 (95% CI 0.63 to 1.12; *P*=.23), indicating no statistically significant difference between groups.

## Discussion

### Principal Findings

This systematic review and meta-analysis synthesized evidence on SMS text message–delivered lifestyle programs for adults with prediabetes, focusing on changes in BMI, weight, waist circumference, HbA_1c_, total cholesterol, and diabetes incidence. Overall, the pooled estimates did not demonstrate statistically significant improvements in these metabolic outcomes compared with standard care, although several individual trials showed favorable trends.

mHealth technologies can extend the reach of prevention programs by lowering barriers related to access, workforce capacity, and cost. SMS text messaging is especially attractive because it is simple, low-cost, and highly scalable [18,29]. For adults with prediabetes—a group often characterized by low disease awareness and variable motivation—SMS text messaging may provide timely prompts for early lifestyle change. However, in contrast to prior meta-analyses reporting benefits of SMS text messaging for smoking cessation, weight loss, cardiovascular risk reduction, or glycemic control in type 2 diabetes [30-37], our results showed null effects on anthropometry and cardiometabolic markers in prediabetes. These differences likely reflect population context and mechanism: approaches successful in patients with established chronic disease may not translate directly to prevention in largely asymptomatic prediabetes.

Several design features may explain the limited impact observed. First, interventions varied widely in duration, message frequency, delivery channel, and content design. Frequencies ranged from multiple messages per week to biweekly contact; the literature is mixed on whether a higher cadence improves outcomes or induces message fatigue, suggesting an outcome- and population-specific optimum. Second, most programs used standardized, nontailored content. Evidence from digital behavior change research indicates that dynamic tailoring—linking message content, timing, and cadence to a person’s current behavior, preferences, and psychosocial state—improves engagement and maintenance [38]. Third, the predominant communication style was unidirectional. Bidirectional designs that solicit brief replies, troubleshoot barriers, and escalate support after nonresponse may enhance accountability and adherence but were rarely implemented. Finally, several trials combined SMS text messaging with additional components (eg, app access, in-person education, or follow-up calls), which improved ecological validity but reduced the ability to attribute effects specifically to SMS text messaging alone and added heterogeneity in behavioral dose and support intensity [39-41].

### Interpreting Null BMI Effects Through Body Composition and Fat Distribution

BMI is an imperfect proxy for metabolic risk because it conflates fat and lean mass and cannot distinguish visceral adipose tissue (VAT) from subcutaneous fat or capture ectopic depots (hepatic or intramuscular fat). Small but meaningful reductions in VAT—or preservation or increases in skeletal muscle mass with modest fat loss—can yield little or no net change in BMI while still improving insulin sensitivity, lipid handling, and inflammatory tone. Early behavior change can also redistribute fat (from visceral to subcutaneous compartments) or prevent sarcopenic weight loss, effects that BMI cannot detect.

In our synthesis, waist circumference similarly showed a precise null effect with relatively narrow CIs; however, centimeter-level changes over short follow-up periods may be below detection thresholds, and meaningful body composition adaptations often require ≥6 to 12 months to manifest. Moreover, adherence decay and metabolic adaptation (reductions in energy expenditure during weight loss) can flatten BMI trajectories when interventions are light touch and largely unidirectional, as seen in most included programs. Therefore, future trials should include body composition outcomes (eg, dual-energy X-ray absorptiometry–derived percent fat and lean mass, VAT area by imaging, and multifrequency bioimpedance) and waist-to-height ratio alongside the BMI.

Regarding publication bias, the small number of studies per outcome precluded formal small-study tests. Notably, several pooled estimates centered closely on the null value with relatively narrow CIs (eg, total cholesterol), suggesting that, at the tested dose and design—generic, 1-way prompts over limited durations—SMS text messaging alone is unlikely to generate the sustained energy deficit and resistance-type stimulus required to materially shift BMI or central adiposity in prediabetes. Thus, the persistence of null results appears plausible even if publication bias might ordinarily favor positive findings.

### Clinical and Public Health Implications

SMS text messaging appears best positioned as a reach-and-reinforcement layer rather than a stand-alone metabolic intervention. In clinical pathways, brief messaging can reinforce clinic-initiated goals, prompt self-monitoring (weight, steps, and dietary logs), and support attendance at higher-intensity services (group classes, dietitian visits, or structured exercise). At the service level, programs should incorporate closed-loop features (eg, automated outreach after nonresponse, brief telecoaching, or referral triggers when weight plateaus) and integrate device-captured data to enable tailoring.

Commissioners planning population strategies should pair SMS text messaging with an effective dose (≥12‐24 weeks), link to evidence-based nutrition and progressive resistance training supports, and evaluate outcomes beyond BMI, including waist-to-height ratio, body fat percentage, VAT surrogates, and glycemic variability. Reporting of engagement analytics (delivery, open, reply, and attrition rates) and behavioral mediators (diet quality, step counts, and resistance training adherence) is essential to clarify mechanisms and optimize deployment.

### Limitations

Several limitations of this study should be acknowledged. Moderate to high heterogeneity was identified in outcomes such as HbA_1c_ levels and diabetes incidence, primarily due to the limited number of available studies (n=3‐4) for these outcomes. Variability in study designs—including differences in intervention duration, SMS text messaging content, and follow-up periods—also posed challenges in synthesizing and comparing results across studies. Due to the small number of studies for certain outcomes, conducting meta-regression analyses and subgroup analyses to adjust for these sources of heterogeneity was not feasible, potentially impacting the reliability of pooled estimates. Assessment of funnel plot asymmetry for publication bias was also precluded due to the limited number of studies.

Furthermore, this meta-analysis did not exclude studies that integrated additional mHealth components, such as mobile apps, alongside SMS text messaging. The inclusion of multicomponent interventions may have introduced bias, limiting the ability to isolate the independent effects of SMS text messaging on clinical outcomes in prediabetes. This review is also limited by its focus on BMI, which may require a longer follow-up duration to detect meaningful changes. Other measures of body composition, such as fat distribution or muscle mass, were not assessed.

### Conclusions

In summary, this systematic review found that lifestyle modification programs using SMS text messaging did not produce significant improvements in BMI among adults with prediabetes compared with standard care. The limited effectiveness observed may reflect variations in intervention design, including content, frequency, and delivery mode, highlighting the need for more optimized, theory-driven messaging strategies.

Future research should focus on developing standardized, tailored, and interactive SMS text messaging interventions with larger sample sizes and longer follow-up periods. Isolating SMS text messaging as a primary intervention component will also be critical to better determine its role in prediabetes management and diabetes prevention efforts.

## Supplementary material

10.2196/78521 Multimedia Appendix 1 Search strategies.

10.2196/78521 Multimedia Appendix 2 Certainty of evidence (GRADE) summary.

10.2196/78521 Multimedia Appendix 3 Data extraction table.

10.2196/78521 Checklist 1 PRISMA checklist.

## Abbreviations

DM: diabetes mellitus
GRADE: Grading of Recommendations Assessment, Development and Evaluation
HbA_1c_: hemoglobin A_1c_
MD: mean difference
MeSH: Medical Subject Headings
mHealth: mobile health
RCT: randomized controlled trial
VAT: visceral adipose tissue

## References

[1] Saeedi P, Petersohn I, Salpea P (2019). Global and regional diabetes prevalence estimates for 2019 and projections for 2030 and 2045: results from the International Diabetes Federation Diabetes Atlas, 9^th^ edition. Diabetes Res Clin Pract.

[2] Magliano D, Boyko EJ (2021). IDF Diabetes Atlas.

[3] Buysschaert M, Medina JL, Buysschaert B, Bergman M (2016). Definitions (and current controversies) of diabetes and prediabetes. Curr Diabetes Rev.

[4] (2024). Prediabetes – your chance to prevent type 2 diabetes. Center for Disease Control and Prevention.

[5] Echouffo-Tcheugui JB, Perreault L, Ji L, Dagogo-Jack S (2023). Diagnosis and management of prediabetes: a review. JAMA.

[6] Galaviz KI, Weber MB, Suvada KBS (2022). Interventions for reversing prediabetes: a systematic review and meta-analysis. Am J Prev Med.

[7] Lambrinou E, Hansen TB, Beulens JW (2019). Lifestyle factors, self-management and patient empowerment in diabetes care. Eur J Prev Cardiol.

[8] Colberg SR, Sigal RJ, Fernhall B (2010). Exercise and type 2 diabetes: the American College of Sports Medicine and the American Diabetes Association: joint position statement. Diabetes Care.

[9] Artinian NT, Fletcher GF, Mozaffarian D (2010). Interventions to promote physical activity and dietary lifestyle changes for cardiovascular risk factor reduction in adults: a scientific statement from the American Heart Association. Circulation.

[10] Saha S, Muradi V, Preethi BL, Kalra P (2021). Assessment of body mass index in prediabetics. Int J Clin Exper Physiol.

[11] Fischer HH, Durfee MJ, Raghunath SG, Ritchie ND (2019). Short message service text message support for weight loss in patients with prediabetes: pragmatic trial. JMIR Diabetes.

[12] Graham SA, Pitter V, Hori JH, Stein N, Branch OH (2022). Weight loss in a digital app-based diabetes prevention program powered by artificial intelligence. Digit Health.

[13] Lim SL, Ong KW, Johal J (2022). A smartphone app-based lifestyle change program for prediabetes (D'LITE study) in a multiethnic Asian population: a randomized controlled trial. Front Nutr.

[14] Wang Y, Xue H, Huang Y, Huang L, Zhang D (2017). A systematic review of application and effectiveness of mHealth interventions for obesity and diabetes treatment and self-management. Adv Nutr.

[15] Iribarren SJ, Cato K, Falzon L, Stone PW (2017). What is the economic evidence for mHealth? A systematic review of economic evaluations of mHealth solutions. PLoS One.

[16] Bootwong P, Intarut N (2022). The effects of text messages for promoting physical activities in prediabetes: a randomized controlled trial. Telemed J E Health.

[17] Hall AK, Cole-Lewis H, Bernhardt JM (2015). Mobile text messaging for health: a systematic review of reviews. Annu Rev Public Health.

[18] Head KJ, Noar SM, Iannarino NT, Grant Harrington N (2013). Efficacy of text messaging-based interventions for health promotion: a meta-analysis. Soc Sci Med.

[19] Lev Arey D, Blatt A, Gutman T (2022). A self-determination theory and acceptance and commitment therapy-based intervention aimed at increasing adherence to physical activity. Front Psychol.

[20] Bol N, Smit ES, Lustria ML (2020). Tailored health communication: opportunities and challenges in the digital era. Digit Health.

[21] Lauffenburger JC, Barlev RA, Sears ES (2021). Preferences for mHealth technology and text messaging communication in patients with type 2 diabetes: qualitative interview study. J Med Internet Res.

[22] Yoon S, Wee S, Loh DH, Bee YM, Thumboo J (2022). Facilitators and barriers to uptake of community-based diabetes prevention program among multi-ethnic Asian patients with prediabetes. Front Endocrinol (Lausanne).

[23] Nanditha A, Thomson H, Susairaj P (2020). A pragmatic and scalable strategy using mobile technology to promote sustained lifestyle changes to prevent type 2 diabetes in India and the UK: a randomised controlled trial. Diabetologia.

[24] Ramachandran A, Snehalatha C, Ram J (2013). Effectiveness of mobile phone messaging in prevention of type 2 diabetes by lifestyle modification in men in India: a prospective, parallel-group, randomised controlled trial. Lancet Diabetes Endocrinol.

[25] Wong CKH, Siu SC, Wong KW, Yu EYT, Lam CLK (2018). Five-year effectiveness of short messaging service (SMS) for pre-diabetes. BMC Res Notes.

[26] Al-Hamdan R, Avery A, Al-Disi D, Sabico S, Al-Daghri NM, McCullough F (2021). Efficacy of lifestyle intervention program for Arab women with prediabetes using social media as an alternative platform of delivery. J Diabetes Investig.

[27] Chung HW, Tai CJ, Chang P, Su WL, Chien LY (2023). The effectiveness of a traditional Chinese medicine-based mobile health app for individuals with prediabetes: randomized controlled trial. JMIR Mhealth Uhealth.

[28] Khunti K, Griffin S, Brennan A (2021). Promoting physical activity in a multi-ethnic population at high risk of diabetes: the 48-month PROPELS randomised controlled trial. BMC Med.

[29] Armanasco AA, Miller YD, Fjeldsoe BS, Marshall AL (2017). Preventive health behavior change text message interventions: a meta-analysis. Am J Prev Med.

[30] Owei I, Umekwe N, Ceesay F, Dagogo-Jack S (2019). Awareness of prediabetes status and subsequent health behavior, body weight, and blood glucose levels. J Am Board Fam Med.

[31] Tam HL, Wong EM, Cheung K, Chung SF (2021). Effectiveness of text messaging interventions on blood pressure control among patients with hypertension: systematic review of randomized controlled trials. JMIR Mhealth Uhealth.

[32] Emeran A, Burrows R, Loyson J, Behardien MR, Wiemers L, Lambert E (2024). The effect of text message-based mHealth interventions on physical activity and weight loss: a systematic review and meta-analysis. Am J Lifestyle Med.

[33] Orr JA, King RJ (2015). Mobile phone SMS messages can enhance healthy behaviour: a meta-analysis of randomised controlled trials. Health Psychol Rev.

[34] Scott-Sheldon LA, Lantini R, Jennings EG (2016). Text messaging-based interventions for smoking cessation: a systematic review and meta-analysis. JMIR Mhealth Uhealth.

[35] Skinner R, Gonet V, Currie S, Hoddinott P, Dombrowski SU (2020). A systematic review with meta-analyses of text message-delivered behaviour change interventions for weight loss and weight loss maintenance. Obes Rev.

[36] Shariful Islam SM, Farmer AJ, Bobrow K (2019). Mobile phone text-messaging interventions aimed to prevent cardiovascular diseases (Text2PreventCVD): systematic review and individual patient data meta-analysis. Open Heart.

[37] Haider R, Sudini L, Chow CK, Cheung NW (2019). Mobile phone text messaging in improving glycaemic control for patients with type 2 diabetes mellitus: a systematic review and meta-analysis. Diabetes Res Clin Pract.

[38] Sahin C, Courtney KL, Naylor PJ, E Rhodes R (2019). Tailored mobile text messaging interventions targeting type 2 diabetes self-management: a systematic review and a meta-analysis. Digit Health.

[39] Wong AK, Wong FK, Bayuo J, Chow KK, Wong SM, Lee AY (2022). A randomized controlled trial of an mHealth application with nursing interaction to promote quality of life among community-dwelling older adults. Front Psychiatry.

[40] Arambepola C, Ricci-Cabello I, Manikavasagam P, Roberts N, French DP, Farmer A (2016). The impact of automated brief messages promoting lifestyle changes delivered via mobile devices to people with type 2 diabetes: a systematic literature review and meta-analysis of controlled trials. J Med Internet Res.

[41] Wong AKC, Bayuo J, Wong FKY (2024). Experiences of receiving an mHealth application with proactive nursing support among community-dwelling older adults: a mixed-methods study. BMC Nurs.

