# Supplementary material for: Effectiveness of Text Messaging Interventions on BMI Among Adults With Prediabetes: Systematic Review and Meta-Analysis
Source: JMIR Mhealth Uhealth. 2026 Apr 30;14:e78521. doi: 10.2196/78521 (PMC13132020; doi:10.2196/78521)
Supplement: Multimedia Appendix 1 [file mhealth-v14-e78521-s001.pdf]

Appendix 1

Searching Strategies

1. PubMed

Searching Strategy	(("Prediabetes"[Mesh]) OR ((prediabetic OR impaired glucose tolerance OR borderline diabetes OR early stage diabetes OR prediabetic state OR prediabetes OR pre-diabetes OR pre diabetes OR prevention of type 2 diabetes OR diabetes prevention OR risk of Type 2 Diabetes OR impaired glucose regulation OR anti-diabetic OR risk of diabetes OR delay type 2 diabetes OR incident diabetes))<br><br>AND<br><br>((Telescopic OR Telemedicine OR Technology-Based OR telecare OR App-Based OR Mobile OR remote intervention OR Internet-Based OR Internet-facilitated OR internet-delivered OR mobile phone messaging OR short messaging service OR text messages OR text messaging service OR text messaging OR sms OR mobile technology OR Telecare OR Telehealth OR telephone OR e-health OR m-health tools OR Technology-Based OR web-based OR Assistive technologies OR Digital OR Smartphone))
Filter Applied	· Results by Year: 2005-2025<br>· Text availability: Full text<br>· Article language: English

2. MEDLINE (via Ebscohost)

Searching Strategy	(MH(Prediabetes) OR (prediabetic OR impaired glucose tolerance OR borderline diabetes OR early stage diabetes OR prediabetic state OR prediabetes OR pre-diabetes OR pre diabetes OR prevention of type 2 diabetes OR diabetes prevention OR risk of Type 2 Diabetes OR impaired glucose regulation OR anti-diabetic OR risk of diabetes OR delay type 2 diabetes OR incident diabetes))<br><br>AND<br><br>((Telescopic OR Telemedicine OR Technology-Based OR telecare OR App-Based OR Mobile OR remote intervention OR Internet-Based OR Internet-facilitated OR internet-delivered OR mobile phone messaging OR short messaging service OR text messages OR text messaging service OR text messaging OR sms OR mobile technology OR Telecare OR Telehealth OR telephone OR e-health OR m-health tools OR Technology-Based OR web-based OR Assistive technologies OR Digital OR Smartphone))
Filter Applied	· Date of Publication: 2005-2025<br>· Source types: Academic Journals<br>· Language: English

3. CINAHL (via Ebscohost)

Searching Strategy	((MH(Prediabetes) OR (prediabetic OR impaired glucose tolerance OR borderline diabetes OR early stage diabetes OR prediabetic state OR prediabetes OR pre-diabetes OR pre diabetes OR prevention of type 2 diabetes OR diabetes prevention OR risk of Type 2 Diabetes OR impaired glucose regulation OR anti-diabetic OR risk of diabetes OR delay type 2 diabetes OR incident diabetes))<br><br>AND<br><br>((Telescopic OR Telemedicine OR Technology-Based OR telecare OR App-Based OR Mobile OR remote intervention OR Internet-Based OR Internet-facilitated OR internet-delivered OR mobile phone messaging OR short messaging service OR text messages OR text messaging service OR text messaging OR sms OR mobile technology OR Telecare OR Telehealth OR telephone OR e-health OR m-health tools OR Technology-Based OR web-based OR Assistive technologies OR Digital OR Smartphone))
Filter Applied	· Date of Publication: 2005-2025<br>· Source types: Academic Journals<br>· Language: English

4. EMBASE

Searching Strategy	((prediabetic OR impaired glucose tolerance OR borderline diabetes OR early stage diabetes OR prediabetic state OR prediabetes OR pre-diabetes OR pre diabetes OR prevention of type 2 diabetes OR diabetes prevention OR risk of Type 2 Diabetes OR impaired glucose regulation OR anti-diabetic OR risk of diabetes OR delay type 2 diabetes OR incident diabetes))<br><br>AND<br><br>((Telescopic OR Telemedicine OR Technology-Based OR telecare OR App-Based OR Mobile OR remote intervention OR Internet-Based OR Internet-facilitated OR internet-delivered OR mobile phone messaging OR short messaging service OR text messages OR text messaging service OR text messaging OR sms OR mobile technology OR Telecare OR Telehealth OR telephone OR e-health OR m-health tools OR Technology-Based OR web-based OR Assistive technologies OR Digital OR Smartphone))
Filter Applied	· Publication Year: 2005-2025<br>· Age: Adult (18-64 years), Aged (65+ years), Very elderly (80+ years)<br>· Publication type: Article

-

5. Cochrane Central Register of Controlled Trials (CENTRAL)

Searching Strategy	((MeSH descriptor: [Prediabetes] explode all trees) OR prediabetic OR impaired glucose tolerance OR borderline diabetes OR early stage diabetes OR prediabetic state OR prediabetes OR pre-diabetes OR pre diabetes OR prevention of type 2 diabetes OR diabetes prevention OR risk of Type 2 Diabetes OR impaired glucose regulation OR anti-diabetic OR risk of diabetes OR delay type 2 diabetes OR incident diabetes))<br><br>AND<br><br>((Telescopic OR Telemedicine OR Technology-Based OR telecare OR App-Based OR Mobile OR remote intervention OR Internet-Based OR Internet-facilitated OR internet-delivered OR mobile phone messaging OR short messaging service OR text messages OR text messaging service OR text messaging OR sms OR mobile technology OR Telecare OR Telehealth OR telephone OR e-health OR m-health tools OR Technology-Based OR web-based OR Assistive technologies OR Digital OR Smartphone))
Filter Applied	· Original publication year: 2005-2025<br>· Trials

6. Web of Science

Searching Strategy	((prediabetic OR impaired glucose tolerance OR borderline diabetes OR early stage diabetes OR prediabetic state OR prediabetes OR pre-diabetes OR pre diabetes OR prevention of type 2 diabetes OR diabetes prevention OR risk of Type 2 Diabetes OR impaired glucose regulation OR anti-diabetic OR risk of diabetes OR delay type 2 diabetes OR incident diabetes))<br><br>AND<br><br>((Telescopic OR Telemedicine OR Technology-Based OR telecare OR App-Based OR Mobile OR remote intervention OR Internet-Based OR Internet-facilitated OR internet-delivered OR mobile phone messaging OR short messaging service OR text messages OR text messaging service OR text messaging OR sms OR mobile technology OR Telecare OR Telehealth OR telephone OR e-health OR m-health tools OR Technology-Based OR web-based OR Assistive technologies OR Digital OR Smartphone))
Filter Applied	· Publication Year: 2005-2025<br>· Document Types: Article<br>· Languages: English
