# Supplementary material for: Effectiveness of Text Messaging Interventions on BMI Among Adults With Prediabetes: Systematic Review and Meta-Analysis
Source: JMIR Mhealth Uhealth. 2026 Apr 30;14:e78521. doi: 10.2196/78521 (PMC13132020; doi:10.2196/78521)
Supplement: Multimedia Appendix 2 [file mhealth-v14-e78521-s002.docx]

**Supplementary Materials**

**Table S1. Summary of Findings (GRADE)**

Certainty of evidence was assessed per GRADE for each meta-analysed outcome.

| **Outcome** | **Effect estimate (follow-up)** | **Participants (studies)** | **Certainty of evidence (GRADE)** | **Reasons for downgrading** |
| --- | --- | --- | --- | --- |
| Body mass index (BMI) | MD −0.17 kg/m² (95% CI −0.85 to 0.25) | 4,102 (7 RCTs) | Low ⬤⬤◯◯ | a (risk of bias), c (imprecision) |
| Body weight | MD −0.46 kg (95% CI −1.74 to 0.83) | NR (5 RCTs) | Low ⬤⬤◯◯ | a (risk of bias), c (imprecision) |
| Waist circumference | MD −0.36 cm (95% CI −1.09 to 0.36) | NR (6 RCTs) | Low ⬤⬤◯◯ | a (risk of bias), c (imprecision) |
| HbA1c | MD −0.05% (95% CI −0.17 to 0.07) | NR (4 RCTs) | Very low ⬤◯◯◯ | a (risk of bias), b (inconsistency), c (imprecision) |
| Total cholesterol | SMD −0.00 (95% CI −0.06 to 0.06) | NR (5 RCTs) | Moderate ⬤⬤⬤◯ | a (risk of bias) |
| Diabetes incidence | OR 0.84 (95% CI 0.63 to 1.12) | 3,515 (3 RCTs) | Very low ⬤◯◯◯ | a (risk of bias), b (inconsistency), c (imprecision) |

**NOTES:**
a. Risk of bias: Several contributing trials were rated “some concerns” or “high risk,” and all had “some concerns” for deviations from intended interventions; these limitations could bias effects toward or away from the null.
b. Inconsistency: Substantial between-study heterogeneity (e.g., HbA1c I²≈89%; diabetes incidence I²≈51%) with non-overlapping CIs suggests true effect variability across studies.
c. Imprecision: Confidence intervals include both important benefit and no effect (and, for diabetes, possible harm), and optimal information size was not met for most outcomes.
NR = not reported in the summary text; insert participant counts from extraction sheets as available.

**Supplementary Methods S1. Certainty-of-Evidence (GRADE) Approach**

Overview.
We evaluated outcome-level certainty using the GRADE framework for each meta-analysed outcome (BMI, body weight, waist circumference, HbA1c, total cholesterol, and diabetes incidence). Because all included studies were randomized controlled trials (RCTs), each outcome started at high certainty and could be rated down across five domains: risk of bias, inconsistency, indirectness, imprecision, and publication bias. No upgrading criteria were applied.

Evidence profile construction.
For each outcome we compiled: pooled effect size (MD, SMD, or OR) with 95% CI, number of participants and trials, heterogeneity statistics (I², χ²), and qualitative features (e.g., outcome measurement consistency, follow-up timing). These informed domain-specific judgments and final ratings (High, Moderate, Low, Very low).

Prespecified decision rules.
• Risk of bias (study limitations): Downgrade one level if ≥30% of information for an outcome came from trials with high overall risk of bias or if most trials had “some concerns” in key domains (e.g., deviations from intended interventions in non-blinded behavioral/digital interventions). Downgrade two levels if the majority of information was from high-risk trials or there was plausible directional bias.
• Inconsistency (heterogeneity): Downgrade one level if I² ≥50% or if CIs show minimal overlap with differing directions/magnitudes of effect without an explained source; downgrade two levels if I² ≥75% with clearly conflicting effects and no credible explanation; no downgrade if I² <50% with broadly overlapping CIs and consistent directions.
• Indirectness (PICO differences): Downgrade one level if populations, interventions, comparators, or outcomes diverged from the review question; no downgrade when trials enrolled adults with prediabetes receiving text-messaging or similar digital support versus usual care/controls with directly relevant outcomes.
• Imprecision (random error): Downgrade one level if the 95% CI crossed the null and encompassed clinically important effects in either direction or if the optimal information size (OIS) was not met; downgrade two levels if CIs were very wide (spanning appreciable benefit and harm) with insufficient information size. For continuous outcomes, we considered whether CIs included changes generally viewed as clinically important (e.g., ~1 kg weight, ~1 cm waist, ~0.3 kg/m² BMI, ~0.3% HbA1c) while recognizing variation across settings.
• Publication bias: With fewer than 10 studies per outcome, we did not perform funnel plots or small-study tests. We did not rate down for publication bias unless there was other evidence suggesting small-study effects or selective reporting (none identified).

Applying the rules to this review.
We downgraded risk of bias by one level for all outcomes due to pervasive “some concerns” and the presence of some “high risk” trials, particularly for deviations from intended interventions. We downgraded inconsistency for HbA1c (I²≈89%) and diabetes incidence (I²≈51%); other outcomes showed low heterogeneity and were not downgraded for inconsistency. We did not downgrade for indirectness. We downgraded imprecision for BMI, weight, waist circumference, HbA1c, and diabetes incidence because the CIs included clinically important benefit and no effect (and for diabetes, possible harm) and OIS was likely unmet; we did not downgrade imprecision for total cholesterol because the pooled SMD CI was narrow and centered near zero. We did not downgrade for publication bias given the small number of studies per outcome and lack of specific signals.

Outputs.
Final certainty ratings and footnoted rationales are presented in Table S1 (Summary of Findings). The GRADE judgments were made independently by two reviewers, with disagreements resolved by discussion; detailed annotations are available upon request.
