# Supplementary material for: Effectiveness of Text Messaging Interventions on BMI Among Adults With Prediabetes: Systematic Review and Meta-Analysis
Source: JMIR Mhealth Uhealth. 2026 Apr 30;14:e78521. doi: 10.2196/78521 (PMC13132020; doi:10.2196/78521)
Supplement: Multimedia Appendix 3 [file mhealth-v14-e78521-s003.pdf]

Appendix 3
Data Extraction Table

Author (Year of publication)	Study location	Number of participants	Study Population	Duration	Intervention Component	Control Group (CG)	Outcome Measures	Result
Al-Hamdan et al. (2021)	Saudi Arabia	N= 120<br><br>IG: n= 83 (GEP: n= 40 WEP: n= 43)<br><br>CG: n= 37<br><br>*<br>GEP: Group education program<br><br>WEP: WhatsApp education program	Inclusion:<br>Saudi women aged 18–60 years diagnosed to have prediabetes (fasting glucose ≥5.6 to ≤6.9 mmol/L)<br><br>Exclusion:<br>Men; women already diagnosed with type 2 diabetes; women receiving antihyperglycemic treatment, antihypertensive or lipid-lowering drugs; pregnant or lactating women; women with known renal, hepatic, pulmonary or cardiac complications; women who were non-active users of android or IOS-based smartphones	6months	<u>App based educational program with Whatsapp SMS support</u><br><br>(a) App +Whatsapp SMS<br><br>(b) WEP participants are required to download "Al-Nahdi Mobile App" an app based educational program about lifestyle modifications emphasizing the importance of weight loss, healthy diet and physical activity. Messages about diet therapy, exercise, general diabetes and prediabetes care knowledge were reviewed by an endocrinologist and sent to the Whatsapp group by the diabetes educator.<br><br>(c) Messages were sent every 2 weeks for 3 months, then continue to follow up until the 6-month period	<u>Usual care</u><br><br>Participants were given lifestyle advice and non-personalized counseling by the assigned primary healthcare center physician. This advice included distribution of translated pamphlets and booklets with information related to 12–14 lifestyle changes from proven effective programs	(1) BMI (kg/m2)<br>(2) HbA1c (%)<br>(3)Weight (kg)<br>(4)Waist Circumference (cm)<br>(5) Total Cholesterol (mmol/L)	(1) BMI (kg/m2)<br>• (IG) mean 29.4; SD 5.1<br>• (CG) mean 31.5; SD 5.7<br><br>(2) HbA1c (%)<br>• (IG) mean 5.3 ; SD 0.51<br>• (CG) mean 5.7; SD 0.49<br><br>(3) Weight (kg)<br>• (IG) mean 73.2; SD 13.8<br>• (CG) mean 75.7; SD 12.4<br><br>(4) Waist Circumference (cm)<br>• (IG) mean 88.8; SD 12.3<br>• (CG) mean 93.4; SD 10.3<br><br>(5) Total Cholesterol (mmol/L)<br>• (IG) mean 5.6; SD 1.4<br>• (CG) mean 5.2; SD 1.1

Bootwong et al. (2022)	Thailand	N= 324<br><br>IG: n= 162<br>CG: n= 162	Inclusion:<br>People whose fasting blood sugar as greater than or equal to 100 and less than or equal to 125 mg/dL; no disorders with cognitive impairment or mental health disorders that have problems reading text mes-sages, and no physical disability that would prevent regular physical activity; ownership of a mobile phone ; availability for the 12-week study duration<br><br>Exclusion:<br>Unable to read and understand mobile phone messages in Thai; people with diabetes, cancer, chronic liver disease and kidney disease	12weeks	(1) Text messages as prompts to encourage physical activity<br><br>(a) Finalized messages were uploaded into a private text messages management program, and sent to participants on a predetermined schedule<br><br>(b) 6 categories related to topics promoting physical activities<br>(i) understanding their own problems, (ii) motivation, (iii) suggesting basic exercise, (iv) mixing exercises in your life, (v) suggesting exercise programs, and (vi) text messaging increased the physical activity time.<br><br>(c) Text messages were sent on 5 days each week (Monday to Friday) for 8 weeks<br><br>(2) Brochure<br><br>(a) Brochure<br><br>(b) Physical activity in prediabetes with instructions. It is meant to introduce intervention participants to physical activity management before receiving text messages on the subject.<br><br>(c) at the beginning of the study	Brochure<br><br>Control group received brochure but no text messages	(1) BMI (kg/m2)<br>(2) Weight (kg)<br>(3) Waist Circumference (cm)	(1) BMI (kg/m2)<br>• (IG) mean 24.2; SD 3.9<br>95% CI: -0.1 (-0.3, 0.2)<br>• (CG) mean 23.7; SD 3.9<br>95% CI: -0.1 (-0.4,0.2)<br><br>(2) Weight (kg)<br>• (IG) mean 60.6; SD 10.0<br>95% CI: -0.2 (-0.7, 0.4)<br>• (CG) mean 58.9; SD 10.4<br>95%CI: -0.4 (-1.1, 0.4)<br><br>(3) Waist Circumference (cm)<br>• (IG) mean 82.5; SD 8.2<br>95% CI: -0.8 (-1.8, 0.2)<br>• (CG) mean 82.2; SD 9.1<br>95% CI: 0.4 (-1.2, 1.9)<br><br>(95% CI: endpoint-baseline)

Chung et al. (2023)	Taiwan	N= 121<br><br>IG: n= 83 (TCM: n= 42; OMG: n= 41)<br><br>CG: n= 38<br><br>*<br>TCM: Traditional Chinese Medicine mHealth group<br><br>OMG: Ordinary mHealth group	Inclusion:<br>Patients who have been diagnosed with prediabetes (according to an HbA1c of 5.7%-6.4% or an FPG level of 100-125 mg/dL); aged 20 years and above; not having cardiopulmonary disease, cancer, or other major diseases; provision of informed consent<br><br>Exclusion:<br>Patients who had used hypoglycemic agents, β-blockers, thiazide diuretics, nicotinic acid, or steroids within the past 3 months were excluded.	16weeks	The ordinary mHealth app<br><br>(a) Personal and group SMS chat room<br><br>(b) The app consists of four modules: health diary, health education, milestone, and chat room which provides information about specific topics such as learning about prediabetes, Dietary Approaches to Stop Hypertension (DASH) diet, and physical activities. The researchers sent text messages to the participants in the personal chat room in both intervention groups, provided feedback on the results, and encouraged participants to share their experiences in the group chat room.<br><br>(c) A text message was sent to the participants to remind them to read the topics every week.	Usual care<br><br>The usual care was 15-20 minutes of health education by family medicine physicians, including disease explanation, healthy diet advice, and exercise encouragement.	(1) BMI (kg/m2)<br>(2) HbA1c (%)	(1) BMI (kg/m2)<br>● (IG) OMG: mean 25.97; SD 5.06<br>● (CG) mean 25.10; SD 3.08<br><br>(2) HbA1c (%)<br>● (IG) OMG: mean 5.94; SD 0.26<br>● (CG) mean 6.02; SD 0.31
Khunti et al. (2021)	England	N=1366<br><br>IG:<br>(1) Walking Away (n=450)<br><br>(2) Walking Away Plus (**n=456**)<br><br>CG: (n=460)	Inclusion:<br>People with plasma glucose/ HbA1c value in the nondiabetic hyperglycaemia range (HbA1c ≥ 42 [6.0], < 48 [6.5] mmol/mol [%]; fasting glucose ≥ 5.5, < 7.0 mmol/l; 2-h post-challenge glucose ≥ 7.8, < 11.1	48months	Walking Away Plus (WAP)<br><br>(a) Face-to-face + mHealth follow up in text messages<br><br>(b) Participants assigned to 3-hours group-based, theory-driven, behavioral intervention addressing knowledge and perceptions of diabetes risk and promoting increased physical activity (up to 3000 steps/day).<br>In addition, participants received text messaging service for setting goals	Leaflet<br><br>Receiving leaflet targeting knowledge of nondiabetic hyperglycaemia and highlighting the importance of physical activity	(1) BMI (kg/m2)<br>(2) HbA1c (%)<br>(3) Weight (kg)<br>(4) Waist Circumference (cm)<br>(5) Total Cholesterol (mmol/L)<br>(6) Number of people developing diabetes	(1) BMI (kg/m2)<br>● (IG) mean 28.6; SD 5.1<br>● (CG) mean 29.2; SD 6.0<br><br>(2) HbA1c (%)<br>● (IG) mean 6.0 ; SD 0.4<br>● (CG) mean 5.9; SD 0.4<br><br>(3) Weight (kg)<br>● (IG) mean 80.8; SD 18.3<br>● (CG) mean 81.8; SD 18.6

			mmol/l) within the last 5 years; aged between 40–74 for White Europeans/ aged 25–74 for those from an ethnic minority; with access to a mobile phone.<br><br>Exclusion:<br>Individuals unable to take part in ambulatory- based activity; were pregnant; diagnosed with diabetes or non-English speakers		and to text back step counts. Automated feedback was then texted to participants with the content tailored to success with achieving goals. Participants also received a further telephone call at six months to review progress.<br><br>(c) Participants receive text messages weekly over the first 6 months, then monthly; Telephone call at six months to review progress			(4) Waist Circumference (cm)<br>• (IG) mean 99.5; SD 13.8<br>• (CG) mean 100.6; SD 14.7<br><br>(5) Total Cholesterol (mmol/L)<br>• (IG) mean 4.8; SD 1.1<br>• (CG) mean 4.8; SD 1.0<br><br>(6) DM Incidence<br>• (IG) n= 41(10.4%)<br>• (CG) n=39 (9.3%)
Nanditha et al. (2020)	India and UK	N=2062<br><br>IG: n= 1031<br>CG: n= 1031	Inclusion:<br>People with prediabetes whose HbA1c ≥42 and ≤47 mmol/mol (≥6.0% and ≤6.4%); No personal history of diabetes or other physical or mental illness<br><br>> For indian participants: including age 35–55 years; having three or more risk factors: BMI ≥23 kg/m2, waist circumference ≥90 cm in men and ≥80 cm in women, first degree family history of type 2 diabetes, history	24months	Regular SMS messages<br><br>(a) Supportive text messages using mobile phone SMS messages<br><br>(b) The messages provided tips, suggestions and positive rein-forcement for healthy behaviors including goal setting, physical activity, dietary planning and personal strategies for lifestyle change. The message content was based on the TTM of behavioral change<br><br>(c) 2-3 text messages per week throughout the 24-month study period.	Usual Care<br><br>Received personalized education and motivation about healthy diet and the benefits of enhanced physical activity at baseline.	(1) BMI (kg/m2)<br>(2) HbA1c (%)<br>(3)Weight (kg)<br>(4)Waist Circumference (cm)<br>(5) Total Cholesterol (mmol/L)<br>(6) Number of people developing diabetes	(1) BMI (kg/m2)<br>• (IG) mean 28.2; SD 4.4<br>• (CG) mean 28.3; SD 4.6<br><br>(2) HbA1c (%)<br>• (IG) mean 6.1; SD 0.5<br>• (CG) mean 6.1; SD 0.5<br><br>(3) Weight (kg)<br>• (IG) mean 77.4; SD 14.7<br>• (CG) mean 78.1; SD 14.9<br><br>(4) Waist Circumference (cm)<br>• (IG) mean 96.2; SD 10.7<br>• (CG) mean 96.3; SD 10.8<br><br>(5) Total Cholesterol (mmol/L)<br>• (IG) mean 5.0; SD 1.0<br>• (CG) mean 5.0; SD 1.0

			of hypertension or prediabetes, or habitual sedentary behaviour<br><br>> For Uk participants: aged 40–74 years without pre-existing diabetes, cardiovascular disease or kidney disease.<br><br>Exclusion:<br>People without smartphone; Patients who already diagnosis with diabetes; Inability to read SMS message					(6) DM Incidence<br><br>● (IG) 216 (21.0%)<br>● (CG) 234 (22.7%)
Ramachandran et al. (2013)	India	N= 537<br><br>IG: n= 271<br>CG: n=266	Inclusion:<br>People with no diabetes (self-reported) or major illness, such as cancer, chronic liver or kidney disease; No physical disability that would prevent regular physical activity; Age 35–55 years; Ownership of a mobile phone and ability to read and understand mobile phone messages in English; a positive family history of type 2 diabetes; BMI of 23 kg/m² or more.<br><br>Exclusion:<br>Recruitment in another trial; Having disorders with cognitive impairment, severe depression or mental imbalance;	24months	<u>SMS Interventions</u><br><br>(a) SMS messages<br><br>(b) Messages contained information about healthy lifestyle, the benefits of physical activity and diet, cues to start physical activity and healthy dietary practices, and strategies to avoid relapse and remain motivated to maintain physical activity and healthy dietary habits.<br><br>(c) Timing (0500–0800 h or 1700–2000 h) and frequency of mobile phone messaging were tailored to the participants' preferences, which were assessed at the 6-monthly visits.	<u>Standard Care</u><br><br>Control group would receive the same personalized education and motivation about healthy lifestyle principles, and written information about diet and physical activity with the intervention group .	(1) BMI (kg/m2)<br>(2)Waist Circumference (cm)<br>(3)Total Cholesterol (mmol/L)<br>(4) Number of People developing diabetes	(1) BMI (kg/m2)<br><br>● IG: mean 25.0, SD 5.5<br>● CG: mean 25.0, SD 5.4<br>● Difference in mean change: -0.05 (95% CI: -0.46 to 0.37)<br><br>(2) Waist Circumference (cm)<br><br>● IG: mean 92.6, SD 7.9<br>● CG: mean 92.6, SD 7.7<br>● Difference in mean change : 0.04 (95%CI: -0.56 to 0.64)<br><br>(3) Total cholesterol (mmol/L)<br><br>● IG: mean 4.9, SD 0.9<br>● CG: mean 4.9, SD 0.9<br>● Difference in mean change:

								0.010 (95%CI: -0.08 to 0.1.)<br><br>(4) DM Incidence:<br>● (IG) n= 50 (18%)<br>● (CG) n= 73 (27%)
Wong et al. (2018)	Hong Kong	N=104<br><br>IG: n=54<br>CG: n=50	Inclusion:<br>People with fasting plasma glucose level of < 7.0 mmol/L or 2-h post-load plasma glucose (2HPPG) of 7.8–11.0 mmol/L after a 75-g glucose load; accessible and receive Chinese messages by mobile phone<br><br>Exclusion:<br>People who had a history of DM; were on medicines known to alter glucose tolerance; were unable to read Chinese characters; refused to take part in study	2years intervention, follow up for 60 months	<u>SMS intervention</u><br><br>(a) SMS via cellular phones<br><br>(b) Text messages including information about diabetes & prediabetes, and lifestyle modification<br><br>(c) Text messages were sent three times a week, once per week and once per month within the first 3 months, the second 3 months, and the subsequent 18 months, respectively.	<u>Booklets</u><br><br>Participants were given booklets with information of prediabetes & diabetes by the research nurse	(1) BMI (kg/m2)<br>(2)Weight (kg)<br>(3)Waist Circumference (cm)<br>(4) Total Cholesterol (mmol/L)	(1) BMI (kg/m2)<br>● (IG) mean 25.14; SD 3.33<br>● (CG) mean 25.97; SD 3.08<br><br>(2) Weight (kg)<br>● (IG) mean 68.76; SD 10.88<br>● (CG) mean 71.33; SD 10.23<br><br>(3) Waist Circumference (cm)<br>● (IG) mean 90.82; SD 8.18<br>● (CG) mean 92.95; SD 7.85<br><br>(4) Total Cholesterol (nmol/L)<br>● (IG) mean 4.96; SD 0.87<br>● (CG) mean 5.13; SD 0.85
